# Expression of the Genes Encoding the Trk and Kdp Potassium Transport Systems of *Mycobacterium tuberculosis* during Growth *In Vitro*

**DOI:** 10.1155/2015/608682

**Published:** 2015-08-17

**Authors:** Moloko C. Cholo, Elizabeth J. van Rensburg, Ayman G. Osman, Ronald Anderson

**Affiliations:** ^1^Department of Immunology, Faculty of Health Sciences, University of Pretoria, Pretoria 0001, South Africa; ^2^Department of Genetics, Faculty of Natural and Agricultural Sciences, University of Pretoria, Pretoria 0001, South Africa

## Abstract

Two potassium (K^+^)-uptake systems, Trk and Kdp, are operative in *Mycobacterium tuberculosis* (*Mtb*), but the environmental factors triggering their expression have not been determined. The current study has evaluated the expression of these genes in the *Mtb* wild-type and a *trk*-gene knockout strain at various stages of logarithmic growth in relation to extracellular K^+^ concentrations and pH. In both strains, mRNA levels of the K^+^-uptake encoding genes were relatively low compared to those of the housekeeping gene, *sigA*, at the early- and mid-log phases, increasing during late-log. Increased gene expression coincided with decreased K^+^ uptake in the context of a drop in extracellular pH and sustained high extracellular K^+^ concentrations. In an additional series of experiments, the pH of the growth medium was manipulated by the addition of 1N HCl/NaOH. Decreasing the pH resulted in reductions in both membrane potential and K^+^ uptake in the setting of significant induction of genes encoding both K^+^ transporters. These observations are consistent with induction of the genes encoding the active K^+^ transporters of *Mtb* as a strategy to compensate for loss of membrane potential-driven uptake of K^+^ at low extracellular pH. Induction of these genes may promote survival in the acidic environments of the intracellular vacuole and granuloma.

## 1. Introduction

Potassium (K^+^) is the most abundant intracellular cation in both prokaryotic and eukaryotic cells reaching concentrations of 100 to 1000 mM inside and 0.1 to 10 mM outside the cell [1–3]. The high intracellular concentrations of the cation are required by cells for diverse cellular processes which include maintenance of turgor pressure, regulation of cytoplasmic pH, osmolarity, transmembrane electrical potential, activation of enzymes, gene expression, and stress responses [1–3].

In order to maintain these high concentrations, most prokaryotes possess several K^+^ influx and efflux pumps, which have varying affinities for the cation and are operative over a wide range of extracellular K^+^ concentrations [4, 5]. In* Mycobacterium tuberculosis (Mtb)*, the causative agent of tuberculosis (TB) disease, only two major K^+^-uptake systems, have been identified, namely, the Trk and Kdp. However, in addition, the genes,* mbtG*,* Rv3200c*, and* Rv3237c*, encode the putative K^+^ channels, L-lysine 6-monooxygenase, a possible transmembrane cation transporter, and a conserved hypothetical protein, respectively [6]. These have been sequenced in the* Mtb* genome, but their roles have not been described [6].

The Trk system is composed of two TrkA proteins, CeoB and CeoC, encoded by two highly homologous operonic genes,* ceoB* and* ceoC* [6]. TrkA is a low-affinity, K^+^-uptake pump, transporting K^+^ at neutral pH under aerobic conditions [7]. In* Escherichia coli*, in which it has been extensively characterized, it is a constitutively expressed, secondary, active K^+^/H^+^ symporter, operating under similar conditions, but ineffective at acidic pH [8].

The second transporter, Kdp, consists of an active KdpFABC element comprising the KdpF, KdpA, KdpB, and KdpC proteins, as well as the two-component response-regulator KdpDE systems comprising the KdpD and KdpE proteins. The two systems are encoded by the* kdpFABC* and* kdpDE* operons, which differ from those of other bacteria, including* E. coli*, being transcribed in opposite directions from each other and separated by an intergenic promoter region of approximately 192 bp [6]. Similar to those of other bacteria, Kdp is an inducible, high-affinity K^+^ transporter functioning as an emergency scavenger when extracellular K^+^ concentrations are low or when the Trk system is either not functioning or absent [7, 9]. It has been characterized as being a primary, active ATP-driven K^+^/H^+^ antiporter [10].

During TB infection, bacteria reside intracellularly and extracellularly in macrophages and necrotic granuloma, respectively, where they are exposed to adverse growth conditions including low pH as well as decreased oxygen and nutrient concentrations [11, 12]. However, the possible involvement of the mycobacterial K^+^ transporters in sustaining growth and survival in hostile environments has not been defined.

In the current study, the roles of the Trk and Kdp systems of* Mtb* during logarithmic growth, as well as the factors that lead to their induction* in vitro*, have been investigated. These include the relative expression of genes encoding the Trk and Kdp systems in the wild-type (WT) and* trk*-gene knockout strain of* Mtb* (expressing the Kdp system only) in relation to rate of growth, uptake of K^+^, extracellular K^+^ concentration, pH level, and membrane potential under aerobic conditions.

## 2. Materials and Methods

### 2.1. Reagents, Primers, and Chemicals

Primer sequences for* trk* and* kdp* target genes, as well as* sigA* used for real-time, reverse-transcriptase polymerase chain reaction (RT-PCR), were designed with Primer 3 Version 0.4.0 [13] and evaluated for dimerization with integrated deoxyribonucleic acid (DNA) technologies (IDT) OligoAnalyzer 3.1 [14]. These primers were synthesized by Inqaba Biotechnology (Pretoria, South Africa). The primer sequences and amplicon sizes of different genes are shown in Table 1.

Rubidium-86 chloride (^86^Rb^+^) 37 MBq was purchased from PerkinElmer Radiochemicals, Boston, MA, USA, and used as a surrogate for measurement of K^+^ uptake by* Mtb* strains [7].

Unless otherwise indicated, all other chemicals were obtained from Sigma Chemical Co. (St. Louis, MO, USA).

### 2.2. Strains and Growth Media

The bacterial isolates used were WT (H37Rv; ATCC 26518) and the* trk*-gene knockout of* Mtb* [7]. The* trk*-gene knockout strain was constructed from the WT strain using homologous recombination following a two-step strategy [7]. Briefly, the* trk*-encoding genes,* ceoB* and* ceoC*, were sequentially mutated in a p2NIL vector and electroporated into the WT strain, resulting in the formation of the single crossover clone, which subsequently lost the plasmid, forming the double crossover colony, referred to as the* trk*-gene knockout strain.

Middlebrook 7H9 broth and 7H10 agar, supplemented with 10% oleic acid, albumin, dextrose, catalase (OADC), and 0.2/0.5% glycerol with/without 0.05% Tween 80, were used as growth media.

### 2.3. Bacterial Culture Preparation

The bacteria were grown in 7H9 broth and incubated at 37°C under stirring conditions to mid-log phase. Cultures were centrifuged at 3500 ×g at 25°C for 10 min and the pellets resuspended in 7H9 broth to an optical density (OD) of 0.6 at 540 nm (equivalent to 10^7^–10^8^ colony-forming units (cfu)/mL). Approximately 10^5^–10^6^ cfu/mL cells were inoculated into 7H9 broth to early-, mid-, and late-log phases, corresponding to ODs of 0.1–0.3, 0.4–0.6, and 2.0–2.3 at 540 nm, respectively.

### 2.4. Gene Expression Using RT-PCR

#### 2.4.1. RNA Isolation, Purification, and Quantitation

Ribonucleic acid (RNA) was isolated using the Trizol method as described previously [15, 16]. Briefly, the bacterial cultures were centrifuged as described above and the cell pellets were resuspended in 1 mL of Trizol (Invitrogen, Carlsbad, CA, USA). The cells were mixed with 0.1 mm zirconia beads (300 *μ*L), lysed for 3 cycles at 45 s each in a Mini-BeadBeater (BioSpec Products, Bartlesville, OK, USA), and centrifuged at 13 000 ×g for 45 s. The supernatants were extracted and mixed with equal volumes of chloroform: isoamyl alcohol (24 : 1 v/v) in a phase-lock gel (Eppendorf, Germany) and centrifuged for 5 min. The recovered supernatants were mixed with isopropanol (600 *μ*L) and incubated overnight at 4°C followed by centrifugation at 13 000 ×g for 20 min at 4°C after which the supernatants were discarded. The RNA pellets were washed once in 70% ethanol (1 mL), air-dried, and treated with DNase I (10 *μ*L RDD buffer, 1 U enzyme; Qiagen, Valencia, CA, USA) for 30 min at 37°C followed by purification using the RNeasy kit (Qiagen) following the manufacturer's instructions. The RNA was eluted in 0.1% diethylpyrocarbonate (DEPC) water and its concentration and purity were determined by* A*_260_/*A*_280_ ratio reading of >1.8 using a NanoDrop spectrophotometer (NanoDrop Technologies, Wilmington, DE, USA). Its integrity was verified electrophoretically on 2% agarose gel in 1% sodium dodecyl sulfate (SDS).

#### 2.4.2. cDNA Synthesis

The complementary DNA (cDNA) was synthesized following a two-step RT-PCR procedure described in the Sigma Enhanced Avian HS RT-PCR kit (Sigma-Aldrich). The annealing mixture for each gene consisting of 100 ng total RNA, 0.5 *μ*M deoxynucleotide triphosphate (dNTP) mixture (dATP, dTTP, dCTP, and dGTP), and 0.5 *μ*M gene-specific reverse primer, made to 10 *μ*L final volume, was incubated at 94°C for 90 s, followed by 65°C for 3 min and 62°C or 57°C for 3 min for the reference or target genes, respectively. The mixture was treated with 0.5 U RNase inhibitor and 0.5 U enhanced avian myeloblastosis virus reverse transcriptase (20 *μ*L) and incubated at 60°C for 30 min and 95°C for 5 min.

#### 2.4.3. Real-Time RT-PCR

The RT-PCR reaction was performed using the LightCycler FastStart DNA Master SYBR Green I kit (Roche Molecular Biochemicals, Johannesburg, South Africa) following the manufacturer's instructions. A 20 *μ*L mixture consisting of 5 ng cDNA, 0.5 *μ*M of each primer (forward and reverse), 4 mM MgCl_2_, and 1X LC FastStart DNA Master SYBR Green I reaction mixture (FastStart Taq DNA polymerase, reaction buffer, and dNTP mixture, SYBR Green I dye, and MgCI_2_) was added to the LightCycler glass capillary tube and heated at 95°C for 10 min followed by amplification for 40 cycles at 95°C for 10 s, 62°C or 57°C for 30 s, and 72°C for 30 s in a LightCycler 2.0 instrument (Roche Molecular Biochemicals). At the end of the amplification reaction, the melting curves of the PCR products were performed at 65°C for 15 s with single and continuous fluorescence measurement at a heating rate of 0.1°C per second and finally cooled at 40°C to check for the specificity of the products using their melting temperatures (Tm) (Table 1). The primer pairs used for amplicon construction for each gene resulted in a quantification cycle (Cq) [17] value of zero or >35 for the nontemplate control.

#### 2.4.4. Relative Quantification Analysis

The expression levels of the individual genes in the mutant relative to those of the WT strain were determined using the 2^−ΔΔCq^, efficiency-corrected, and the LightCycler methods [18–21]. The amplification efficiencies were 2 for all the genes using the 2^−ΔΔCq^ method, while those used in efficiency-corrected and LightCycler methods were derived from generated calibration curves for each gene (LightCycler version 4.0). The reference gene was the* sigA* which, based on the Cq values, demonstrated a constant expression throughout the log phase [22, 23].

#### 2.4.5. Absolute Quantification Analysis

The calibration curves for each gene, including* sigA*, were generated with genomic standards prepared in 5-fold dilutions containing from 0.0512 to 10^5^ *μ*g/mL DNA per reaction for each gene.

### 2.5. Bacterial Growth

Approximately 10^5^–10^6^ cfu/mL cells were inoculated into 7H9 broth and incubated at 37°C under stirring conditions for nine days. The cultures were sampled daily, diluted, and plated onto 7H10 agar medium and incubated for three weeks until the colonies appeared. The number of colonies was then counted and compared between the two strains.

### 2.6. Rubidium Uptake

^86^Rb^+^ was used as a surrogate tracer for the determination of K^+^ uptake by bacteria. Cells grown in 7H9 broth were harvested, washed, and resuspended in glucose-, K^+^- and Na^+^-free (KONO: 46 mM disodium hydrogen orthophosphate, 23 mM sodium dihydrogen orthophosphate, 0.4 mM magnesium sulphate, and 0.6 mM ferrous sulphate) buffer (pH 7.4). Approximately 10^6^ cfu/mL cells, prewarmed at 37°C, were treated with 1 mCi/L ^86^Rb^+^ in 2 mL KONO buffer containing 22 mM glucose and incubated at 37°C for 90 min. Cells were washed with phosphate-buffered saline (PBS) and lysed with 0.4 mL 5% trichloroacetic acid (TCA). The radioactive content of the lysate was measured using liquid scintillation counter as previously described [7, 24].

### 2.7. Measurement of pH and Potassium Concentration of the Growth Medium

Bacterial cultures were centrifuged and the supernatants were decontaminated by heating at 95°C for 60 min. The pH of the supernatants was measured directly in the undiluted samples using the CRISON micropH2001 pH meter (Lasec, Johannesburg) and the K^+^ concentrations were measured in the diluted samples by indirect potentiometry utilizing a K^+^ selective electrode in conjunction with a Na^+^ reference electrode (Synchron LX System, Beckman Coulter, Ireland Inc., Gateway, Ireland). In both cases, the bacteria-free 7H9 broth processed as above, as well as unprocessed growth medium, served as control.

### 2.8. Effects of Alterations in Extracellular pH on Gene Expression, ^86^Rb^+^ Uptake, Membrane Potential, and Growth Rates

For these assays, the bacteria, grown in 7H9 broth to mid-log phase (prior to the decrease in extracellular pH and induction of the genes encoding the K^+^ transporters), were washed once and resuspended in Sauton broth, KONO buffer, or indicator-free Hanks balanced salt solution according to the assays being performed, with the pH adjusted to 5.5, 6, 6.5, and 7 with 1 N hydrochloric acid/sodium hydroxide (HCl/NaOH). Similar procedures were followed as described earlier with some minor modifications. Gene expression was performed on RNA isolated from cells grown in pH-adjusted fresh Sauton broth for 24 hours before harvesting. The rate of growth was determined by inoculating the bacteria, resuspended in pH-buffered Sauton broth into fresh culture media and incubated for 14 days under aerobic conditions. The cultures were sampled and plated on Day 14. ^86^Rb^+^ uptake was determined using bacteria suspended in pH-adjusted KONO buffer containing ^86^Rb^+^ and glucose.

Membrane potential was determined using bacteria (10^8^ cfu/mL) suspended in pH-adjusted Hanks balanced salt solution (3 mL) in the presence of the membrane potential-sensitive fluorescent dye, dipentyloxacarbocyanine (di-O-C5(3), 400 nM), at 37°C for 5 min. Alterations in fluorescence intensity were monitored using a microplate luminescence spectrophotometer (LS 50: PerkinElmer) with excitation and emission wavelength settings of 460 and 510 nm, respectively.

### 2.9. Statistical Analysis

All statistical analyses were carried out with the GraphPad Instat program version 3. The paired Student *t*-test was used to analyze the results for gene expression, while those for ^86^Rb uptake, rates of growth, extracellular pH and K^+^ concentration, and membrane potential were analyzed using an unpaired nonparametric, two-tailed test. The results are expressed as the means ± standard error of mean (SEM) and the significance levels were taken at a *p* < 0.05.

## 3. Results

### 3.1. The Relative Expression of the* trk* and* kdp* Genes

The expression levels of the individual genes in the* trk*-gene knockout relative to those of the WT strain were determined using relative quantification analysis. The Cq values of the* sigA* reference gene were different throughout the log phases, but comparable between the two strains. At the early- and mid-log phases the Cq value for both strains was 22, and as the bacteria progressed into the late-log phase, the values were 18.66 and 18.32 for the WT and* trk*-gene knockout strains, respectively.

The efficiencies of calibration curves of the target and* sigA* genes were between 1.8 and 2.3 (SD > 0.05), with the three methods of analysis generating comparable data. As the efficiency values were close to 2.0, the 2^−ΔΔCq^ method was adopted [18–21]. The results are shown in Table 2. Mutations of the* ceoB* and* ceoC* genes in the* trk*-gene knockout strain were confirmed by melting curve analysis, resulting in nonspecific fragments. The magnitudes of expression of the* kdp* genes in the WT and* trk*-gene knockout strains throughout the log phase were comparable, with the exceptions of* kdpE* and* kdpF*, the levels of which were moderately increased in the mutant.

### 3.2. Absolute Expression of* trk* and* kdp* Genes

To characterize the expression of individual genes in each strain at the various stages of growth, the absolute expression values of each gene were extrapolated from individual calibration curves. These results are shown in Table 3. The magnitudes of expression of the* sigA* housekeeping gene, during early- and mid-log phases, were comparable between the two strains and were considerably higher than those of the K^+^-uptake encoding genes. The expression levels of the individual K^+^-transporter-encoding genes were also comparable within and between the two strains. The low levels of all the K^+^-uptake encoding genes indicate that these genes are not upregulated during these growth phases and that the observed expression levels probably represent their basal expression. As the growth of the organisms reached the late-log phase, however, the expression levels of all genes, including* sigA*, were elevated, with the highest expression of the* kdp* genes observed in the* trk*-gene knockout strain. The relative fold increase of these K^+^-uptake genes, as ratios of late-log phase in relation to early-log phase for both strains, is shown in Table 4. These fold increases of all the genes indicate that as the bacteria enter into late-log phase, there are environmental factors in the cultures that lead to the upregulation of these genes. However, the fold increase of the* sigA* gene in both strains during late-log phase, in comparison to the K^+^-uptake encoding genes, was very low, indicating that the change in expression of the* sigA* gene in both strains throughout the log phase of growth is minimal. In addition, the induction of the* kdp* genes in the WT strain was low in relation to that of the mutant indicating that the Kdp system is repressed in the presence of the functional Trk system.

Gene expression levels calculated as a ratio of the mid- and late-log phases of growth relative to the early-log phase for each gene for each strain are shown in Table 4. Using this format of presentation of data, it is clear that there is a significant upregulation of expression of all the K^+^-related transport genes in the WT strain, especially* ceoB*,* kdpC*, and to a lesser extent* kdpB* and* kdpE*, all of these ratios being much higher than that of the* sigA* gene at the late-log phase. Likewise, the ratios for expression of the* kdp* genes in the mutant strain at the late-log phase were considerably elevated both in relation to* sigA* and the corresponding* kdp* genes in the WT strain, with the* kdpC* and the* kdpB* genes being particularly highly expressed. In addition, the expressions of* kdpA*,* kdpB*, and* kdpC* genes were of a comparable ratio of 1 : 1 : 1 during this growth phase within each strain.

### 3.3. Rates of Growth

The rates of growth of the two strains were comparable during the early- and mid-log phases, but the* trk*-gene knockout mutant reached the late-log phase earlier than the WT strain. We have previously found that this mutant strain has a faster rate of growth than the WT when using a linear graph presentation [7]. The results are represented as cfu/mL ± SEM and are shown in Table 5. The inoculum sizes at Day 0 were comparable for both strains and were 2 × 10^6^ ± 2 × 10^5^.

### 3.4. ^86^Rb^+^ Uptake

**Figure 1:**
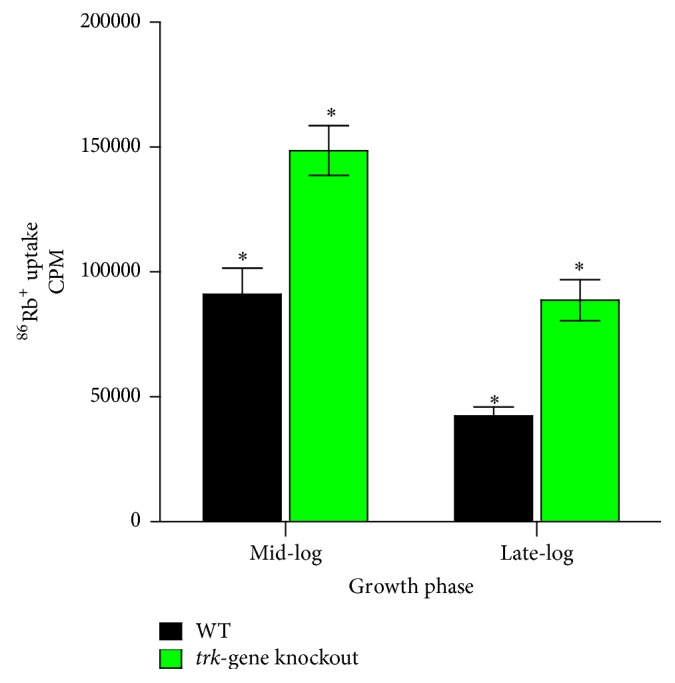
^86^Rb^+^-uptake efficiencies of the WT and* trk*-gene knockout strains at the mid- and late-log phases. The results are of three separate experiments with a total of 10–15 replicates for the mid- and late-log phases, respectively. Statistical significance was determined between the two strains within each growth phase and for each strain between the two growth phases. ^*∗*^ *p* < 0.05. The absolute radioactive cpm values were 91041 ± 10465 and 148615 ± 9918 and 42431 ± 3447 and 88819 ± 8178 for WT and* trk*-gene knockout strains at mid- and late-log phases, respectively.

The efficiencies in K^+^ uptake of the two strains during the mid- and late-log phases were determined by measuring the absolute uptake of ^86^Rb^+^ by the strains during these growth phases. The results are represented as the mean radioactive counts per minute (cpm) values ± SEM and are shown in Figure 1. During both growth phases, the ^86^Rb^+^-uptake efficiencies of the* trk*-gene knockout strain were higher than those of the WT strain. However, the K^+^-uptake efficiencies of both strains were reduced as the bacteria entered the late-log phase.

### 3.5. Extracellular pH and Potassium Concentration

To characterize the factors that lead to decreased efficiency of uptake of K^+^ and upregulation of the K^+^-uptake genes, presumably as a compensatory mechanism, the possible influences of alterations in extracellular pH and the extracellular K^+^ concentration during the three log phases of growth were evaluated. In the case of both strains, the extracellular pH values of the culture media were comparable to those of bacteria-free media (pH 6.8 ± 0.01) at the early-log stage, increased slightly during mid-log (pH 6.9 ± 0.01), and decreased at the late-log phase (pH 6.5 ± 0.01).

With respect to extracellular K^+^ concentrations in the growth media, no significant decreases were detected during the three log phases for both strains, probably reflecting the high K^+^ concentrations of the growth media, as well as possible recycling of K^+^ by the bacteria during growth. The concentrations were 13.3 ± 0.05 mM for the bacteria-free controls, while the paired values for the WT and* trk*-gene knockout strains were 13.4 ± 0.05 and 13.2 ± 0.05, 13.3 ± 0.15 and 13.2 ± 0.15, and 13.5 ± 0.5 and 13.6 ± 0.25 mM during the early-, mid-, and late-log phases, respectively.

### 3.6. Effect of Manipulation of Extracellular pH on Gene Expression, ^86^Rb^+^ Uptake, Membrane Potential, and Rate of Growth

Because the increase in the magnitude of gene expression of the K^+^ transporters and the decrease in K^+^ uptake (Section 3.2) during late-log phase coincided with a decrease in pH of the growth medium, the possible role played by alterations in extracellular pH on the magnitudes of expression of the K^+^-uptake genes in relation to rate of growth, uptake of ^86^Rb^+^, and membrane potential was investigated in a series of short-duration experiments.

**Figure 2:**
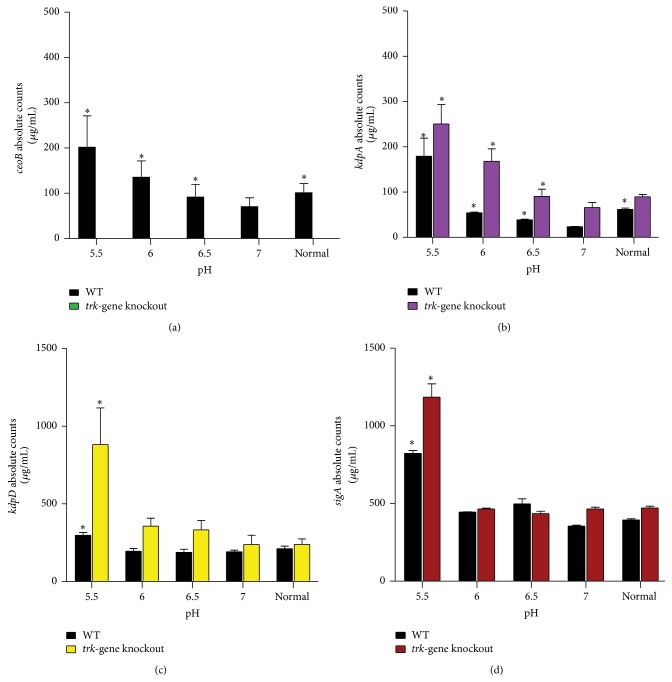
The absolute expression of the* trk* and* kdp* genes in the WT and* trk*-gene knockout cells at mid-log phase suspended in growth medium of varying pH. (a), (b), (c), and (d) represent the absolute expression (*μ*g/mL) of the* ceoB*,* kdpA*,* kdpD*, and* sigA* genes, respectively. Normal represents bacteria cultured in 7H9 broth without adjustment of the pH. The results are of three experiments in duplicate for each gene. Statistical significance was determined in relation to the absolute quantity of each gene at pH 7. ^*∗*^ *p* < 0.05.

For these experiments, based on simultaneous expression of genes from the same operons (Table 3), gene expression was evaluated on one gene of each operon [7] with* sigA* included as a reference gene. All genes, including the* sigA*, were significantly upregulated at pH 5.5 and highest in the* trk*-gene knockout strain. The magnitudes of expression of the* ceoB* and* kdpA* genes were pH-dependent being inversely related to decreasing extracellular pH. Relative to the mutant strain, however, induction of the* kdpA* and* kdpD* genes was less significant in the WT strain (Figure 2). These results indicate that both K^+^-uptake systems are induced at pH 5.5. However, as the pH increases from 6 to 6.5 the expression of the* trk* genes decreases significantly, while the* kdp* genes remain suppressed in the presence of functional Trk system.

**Figure 3:**
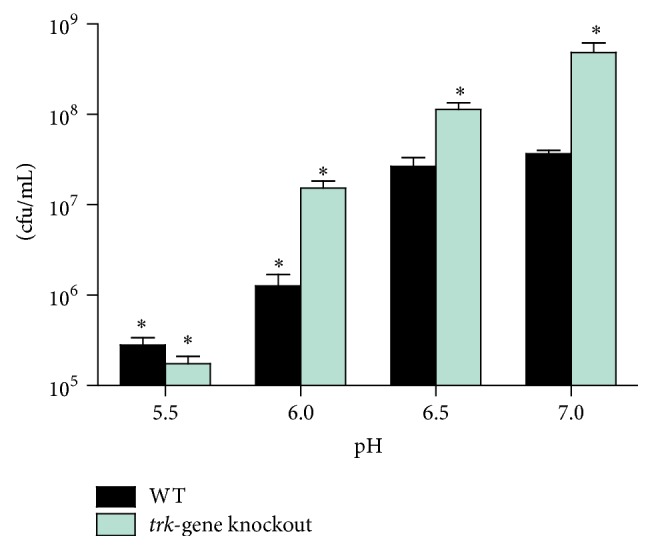
The rates of growth of the WT and the* trk*-gene knockout strains at varying extracellular pH levels grown for 14 days in pH-buffered Sauton media. The results are of six replicates from three experiments represented as colony-forming units/mL ± SEM. The cfu/mL at Day 0 were 5.5 × 10^5^ ± 1.07 × 10^5^ and 4.6 × 10^5^ ± 8 × 10^4^ for the WT and* trk*-gene knockout strains, respectively. At Day 14, cfu/mL were 2.8 × 10^5^ ± 5.4 × 10^4^,  1.2 × 10^6^ ± 4.3 × 10^5^, 2.7 × 10^7^ ± 6.7 × 10^6^, and 3.7 × 10^7^ ± 3.3 × 10^6^ and 1.8 × 10^5^ ± 3.4 × 10^4^, 1.5 × 10^7^ ± 2.8 × 10^6^, 1.1 × 10^8^ ± 2.1 × 10^7^, and 4.9 × 10^8^ ± 1.4 × 10^8^ for growth in pH 5.5, 6.0, 6.5, and 7.0 for WT and* trk*-gene knockout strains, respectively.

**Figure 4:**
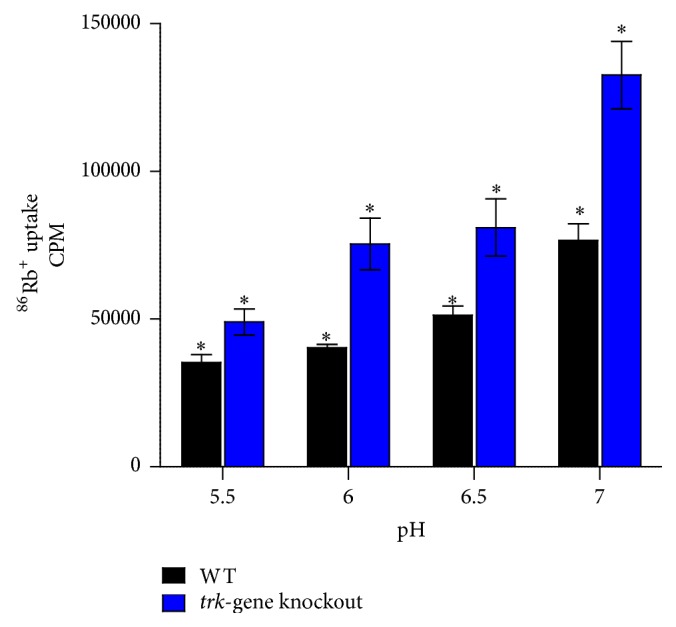
The effects of varying the extracellular pH on the ^86^Rb^+^-uptake efficiencies of the WT and* trk*-gene knockout strains. The results are of three separate experiments with duplicate measurements for each system in each experiment. ^*∗*^ *p* < 0.05.

**Figure 5:**
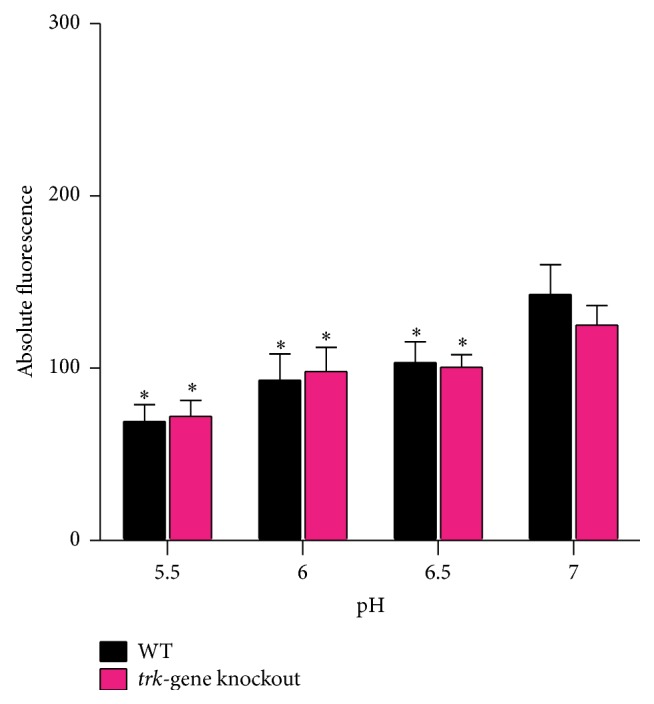
The membrane potential values of  WT and* trk*-gene knockout strains at varying extracellular pH levels. The results represent nine replicates from three experiments represented as absolute fluorescence ± SEM. Statistical significance was determined in relation to membrane potential observed at pH 7 for each strain and between the strains at each pH level. ^*∗*^ *p* < 0.05.

In the case of both strains upregulation of gene expression coincided with decreases in the rate of growth, uptake of ^86^Rb^+^, membrane potentials, and extracellular pH which dropped to 5.5 (Figures 3, 4, and 5).

Both strains failed to grow at pH 5.5 (Figure 3). The numbers of bacteria were 5.5 × 10^5^ ± 1.1 × 10^5^ and 4.6 × 10^5^ ± 8 × 10^4^ cfu/mL ± SEM at Day 0 for the WT and* trk-*gene knockout strains, respectively, with corresponding decreases to 2.8 × 10^5^ ± 5.4 × 10^4^ and 1.8 × 10^5^ ± 3.4 × 10^4^ cfu/mL ± SEM on Day 14. As the two K^+^-uptake systems are highly induced at pH 5.5 (Figure 2), their induction illustrates that these systems are utilized by the bacteria for their survival at this pH. As the extracellular pH increased from 6.0 to 7.0, both strains showed high rates of growth, resulting in at least a 10-fold increase in the number of bacilli in the case of the mutant strain relative to the WT at each pH level.

The decrease in K^+^ uptake observed with both strains was pH-dependent being most evident in the case of the* trk*-gene knockout strain (Figure 4).

The membrane potentials of the two strains were comparable at every pH level decreasing in parallel as the pH dropped to 5.5, showing that the membrane potentials are not affected by the presence of Trk system at these pH levels but rather by a change in extracellular pH (Figure 5).

## 4. Discussion

The results of the current study for the WT strain of* Mtb* demonstrate that the expression levels of the genes encoding the Trk and Kdp K^+^-uptake systems of* Mtb* are extremely low relative to those of the housekeeping gene,* sigA*, during the early- and mid-log phases of bacterial growth. Likewise, in the* trk*-gene knockout strain in which expression of both* ceoB* and* ceoC* was barely detectable, the levels of expression of the* kdp* genes were low and comparable to those of the WT strain. These expression levels suggest that, during these phases of growth in artificial growth medium with a high extracellular K^+^ concentration and neutral pH, utilization of the Trk and Kdp systems is minimal with nonspecific membrane potential-driven uptake of the cation probably predominating, thereby enabling conservation of energy and optimal growth [25]. Alternatively, basal, homeostatic activity of the K^+^ transporters may suffice during the early stages of growth.

However, when the bacteria entered the late-log phase of growth striking increases in the levels of expression of both the* trk* and* kdp* genes were noted in the WT strain, which were disproportionately higher than those observed for* sigA*, the respective fold increases being 188, 230, and 77/75 for* ceoB*,* kdpC*, and* kdpB/kdpE*. In the case of the* trk*-gene knockout, a similar pattern in the expression of the* kdp* genes comparable to that of the WT strain was observed, the corresponding fold increases in the* kdpC* and* kdpB* genes being 950 and 539. Presumably the much higher levels of gene expression of the* kdp* genes in the* trk-*gene knockout relative to the WT strain reflect a compensatory strategy to ensure efficient uptake of K^+^ [7].

This pattern in fold increase in* kdp* gene expression has recently been shown for the synthesis of the Kdp subunits of* E. coli* during K^+^-limiting conditions [26]. However, the increased level of expression of the Trk and Kdp systems in* Mtb* during the late-log phase of growth observed in the current study could not be attributed to depletion of extracellular K^+^ since the concentration of the cation in the growth medium was essentially unchanged throughout the log phase of growth. These results are consistent with the existence of additional stressors, such as the decrease in the pH of the growth medium in the late stages of growth that may trigger the upregulation of the K^+^-uptake genes.

This apparent relationship was addressed in a separate series of experiments in which the effects of decreasing the extracellular pH of the cell-suspending medium using bacteria harvested at the mid-log phase of growth, prior to the decrease in extracellular pH and upregulation of the K^+^ transport genes, on gene expression, rate of growth, uptake of K^+^, and membrane potential were investigated. In these experiments decreasing the extracellular pH resulted in a concomitant drop in membrane potential, uptake of K^+^, and rate of growth in the setting of upregulation of gene expression, consistent with a mechanistic interrelationship between these events. To summarize, disruption of the membrane potential at low extracellular pH attenuates membrane potential-driven uptake of K^+^, necessitating induction of the genes encoding the active K^+^-uptake systems to enable survival. A similar relationship has also been described in* M. smegmatis* [27]. In addition, other types of microorganism which compensate for decreased K^+^ uptake at low extracellular pH include* Streptomyces lividans*, which possesses a pH-gated K^+^ channel [28], and the Kup transporter of* E. coli*, which functions at low pH [8], as well as the K^+^/H^+^ antiporters operative in most Gram-negative bacteria [29].

In these bacteria deletion of the major K^+^-uptake systems (Trk, Kup, and Kdp) resulted in low membrane potential and poor K^+^ uptake, which occurred concomitantly with failure of maintenance of intracellular pH of 7.4 to 7.8 and attenuation of growth at extracellular acidic pH (pH below 4.5) [17, 27, 30, 31]. At this pH, the growth of* Mtb* is arrested, resulting in a nonreplicating dormant state [17] However, at neutral pH, both cytoplasmic pH and bacterial growth rates are unaffected by the presence of the K^+^-uptake systems, illustrating that transportation of K^+^ is independent of the major K^+^-uptake systems at this pH level [27, 31] while at alkaline pH (pH higher than 8), the proton-importing ATP synthase F_1_F_0_ is induced, minimizing the exportation of protons from the cytoplasm [32]. Together with our study, these findings indicate that growth of bacteria at acidic or alkaline pH induces K^+^-uptake genes, which are necessary for the maintenance of internal pH homeostasis and survival of bacteria in these environments [32, 33].

During active disease,* Mtb* bacilli are able to survive in acidic environments, found in macrophage vacuoles and necrotic granuloma [11, 12, 34]. The induction of both the Trk and Kdp systems in acidic environments in the current study indicates that these systems may be required for bacterial adaptation at low pH levels. Following uptake of* Mtb* by macrophages, the phagosome acidifies to pH 6.0, thereafter rising to pH 6.5 within a few hours, enabling the bacteria to survive and multiply [11, 12, 35, 36]. Based on similarities to the TrkA system of* E. coli*, which possesses an NAD^+^-binding motif [37], it is possible, though unproven, that the Trk system of* Mtb*, which has been implicated in intracellular survival [8, 38], may function as a K^+^/H^+^ symporter, increasing vacuolar pH. If indeed operative in the intravacuolar milieu, such a mechanism would be expected to interfere with phagosome maturation, thereby promoting intracellular survival. In this growth environment at these pH levels, the Kdp system is likely to be suppressed. However, other factors, such as low K^+^ concentrations, exist in the macrophages that lead to the induction of this K^+^-uptake system [39]. In the granuloma, where the pH ranges between 5.0 and 5.5 [40, 41], both K^+^-uptake systems may be required.

## 5. Conclusion

According to the gene expression data, the findings of the current study demonstrate that the Trk and Kdp systems of* Mtb* function at basal levels when conditions, especially high extracellular K^+^ concentration and neutral pH, favour membrane potential-driven uptake of K^+^. However, at low extracellular pH, uptake of K^+^ may become increasingly dependent on induction of both the Trk and Kdp systems as a survival strategy in hostile environments.

## Figures and Tables

**Table 1:** Primers for expressed genes in WT and *trk*-gene knockout mutant strains of *Mtb*.

Gene name	Forward primer (bp)	Reverse primer (bp)	Fragment length^a^	Tm^b^
*ceoB* ^c^	CGG CGA CAA CTC CAA CAT (18)	CGG CAC ACC GAA GGT TTC (18)	58	86.2
*ceoC* ^c^	CTG CTG GAG TCG ATT CAC CT (20)	ACC GCG AAT TCG GTC TTG (20)	107	88.9

*kdpA*	GAT TGA ACG GCC TAC TGG TC (20)	CTG GAT CTT CTT GCC GAG AT (20)	95	87.5
*kdpB*	CTG GGC TGA CGA TCA TCT TT (20)	CGT GGT CGG AAT GAG ACA C (19)	122	90.6
*kdpC*	TAC CGC AAG GAA AAC AAT CTG (21)	GGC ATT GAC CAC CGA TATG (19)	102	90.2
*kdpF*	AAC ATC GTC GGG TTG GTG (18)	GCG AAT AGG AAC GCC ATT AG (20)	50	83.7

*kdpD*	AGT CCA TCG ACC AAC TCA CC (20)	CAC CGC TTC CTC CAG GTA T (19)	110	91.4
*kdpE*	TGG AAT GGA CGA GTT TCT GG (20)	*GAC GGT GAA *TGA ATC GGT TT (20)	103	91.4

*sigA*	CAA GGA CGC CGA ACT CAC (18)	CTT GCC GAT CTG TTT GAG GT (20)	64	87.4

^a^Size of the amplicon synthesized by RT-PCR in base pair (bp).

^b^Melting temperature derived from melting curve.

^c^Gene encoding Trk system.

**Table 2:** Expression of the *trk* and *kdp* genes in the *trk*-gene knockout strain of *Mtb* relative to those of the WT.

Gene name	Early-log ratio (2^−ΔΔCq^)^a^ ± SEM	CI^b^	Mid-log ratio (2^−ΔΔCq^)^a^ ± SEM	CI^b^	Late-log ratio (2^−ΔΔCq^)^a^ ± SEM	CI^b^
*ceoB*	0.02 ± 0.01	0.01–0.03	0.016 ± 0.001	0.013–0.02	0.0003 ± 0.001	0.0002–0.00048
*ceoC*	0.01 ± 0.01	0.001–0.01	0.003 ± 0.001	−0.001–0.01	0.002 ± 0.0001	−0.0007–0.01

*kdpA*	1.23 ± 0.11	0.95–1.52	1.03 ± 0.02	0.99–1.07	1.10 ± 0.09	−0.81–1.39
*kdpB*	1.16 ± 0.10	0.9–1.44	1.54 ± 0.16	1.13–1.96	1.30 ± 0.21	0.76–1.85
*kdpC*	1.37 ± 0.14	1.03–1.72	1.00 ± 0.12	0.61–1.23	1.28 ± 0.26	0.62–1.94
*kdpF*	0.82 ± 0.27	0.13–1.51	2.15 ± 0.63	0.52–3.77	1.13 ± 0.18	0.66–1.59

*kdpD*	0.76 ± 0.12	0.48–1.05	0.65 ± 0.13	0.32–0.98	1.39 ± 0.48	0.15–2.63
*kdpE*	1.29 ± 0.19	0.82–1.77	1.84 ± 0.46	0.65–3.02	1.63 ± 0.26	0.96–2.3

^a^Ratio of quantification using delta delta Cq method [13, 14].

^b^Confidence interval.

**Table 3:** Absolute expression of the *trk* and *kdp* genes in WT and *trk*-gene knockout strains of *Mtb*.

Gene name	H37Rv (*μ*g/mL)			*trk*-gene knockout (*μ*g/mL)		
	Early-log ± SEM	Mid-log ± SEM	Late-log ± SEM	Early-log ± SEM	Mid-log ± SEM	Late-log ± SEM
*ceoB*	16.3 ± 6.6	14.7 ± 1.1	3071.0 ± 602.1	0.43 ± 0.1	0.49 ± 0.1	2.34 ± 0.7
*ceoC*	25.7 ± 6.5	25.8 ± 1.3	653.8 ± 68.6	0.13 ± 0.10	0.01 ± 0.01	2.11 ± 0.7

*kdpA*	22.5 ± 8.7	16.4 ± 3.2	618.8 ± 115.7	22.5 ± 5.2	14.7 ± 4.3	3410.6 ± 1192.0
*kdpB *	6.7 ± 3.0	8.2 ± 1.1	519.9 ± 84.4	6.5 ± 2.6	9.2 ± 1.0	3452.3 ± 1266.4
*kdpC *	3.4 ± 1.2	5.4 ± 0.5	787.3 ± 103.3	3.5 ± 0.9	3.3 ± 0.8	3332.2 ± 1109.1
*kdpF*	28.0 ± 9.1	19.6 ± 8.6	1523.8 ± 180.2	11.3 ± 0.5	15.1 ± 1.2	2640.8 ± 648.9

*kdpD*	38.4 ± 5.8	40.2 ± 3.0	1539.5 ± 193.0	34.6 ± 3.0	33.6 ± 5.2	4672.9 ± 1452.0
*kdpE*	9.5 ± 1.7	9.1 ± 2.0	716.8 ± 100.7	10.6 ± 0.4	9.3 ± 1.6	2776.6 ± 805.3

*sigA*	254.5 ± 45.3	208.0 ± 8.1	1088.6 ± 68.2	249.5 ± 36.0	201.6 ± 10.6	2545.8 ± 728.2

**Table 4:** Ratio of gene expression of both strains of *Mtb* during mid- and late-log phases in relation to the corresponding early-log phase.

Gene name	H37Rv		*trk*-gene knockout	
	Mid-log	Late-log	Mid-log	Late-log
*ceoB*	0.9	188.8	NA^a^	NA^a^
*ceoC*	1.0	25.4	NA^a^	NA^a^

*kdpA*	0.7	27.5	0.7	151.4
*kdpB *	1.2	77.7	1.4	534.4
*kdpC *	1.6	235.0	0.9	952.1
*kdpF*	0.7	54.5	1.3	233.7

*kdpD *	1.1	40.1	1.0	135.3
*kdpE*	1.0	75.7	0.9	262.4

*sigA*	0.8	4.3	0.8	10.2

^a^Not available since the expression levels in the mutant for these two genes were not detectable.

**Table 5:** Rate of growth of both strains of *Mtb*.

Growth phase	ODs	Number of colonies cfu/mL ± SEM	Days achieved
Early-log	0.1–0.3	6.0 × 10^6^ ± 6.2 × 10^5^–9.5 × 10^7^ ± 2.1 × 10^7^	1–3
Mid-log	0.4–0.6	6.8 × 10^8^ ± 3.6 × 10^7^–7.0 × 10^10^ ± 1.7 × 10^9^	4-5
Late-log	2–2.3	5.0 × 10^11^ ± 7.0 × 10^9^–2.0 × 10^14^ ± 8.0 × 10^12^	8-9
